# Epidemiology of Respiratory Adenovirus Infections Among Hospitalized Children in Hainan Province, China, 2021–2024

**DOI:** 10.3390/pathogens14121246

**Published:** 2025-12-05

**Authors:** Xiangyue Zeng, Shengjie Shi, Mengchun Chen, Xiaoqi Weng, Jiaoling Wen, Xiucheng Fu, Jing An, Peizhen Zhao, Meng Chang

**Affiliations:** 1 Department of Clinical Laboratory, Center for laboratory Medicine, Hainan Women and Children’s Medical Center, Haikou 570206, China; zxy865673000@163.com (X.Z.); 15874843023@163.com (S.S.); chenmengchun7001@163.com (M.C.); wxq2462156503@163.com (X.W.); 13570648936@163.com (J.W.); m18976448738@163.com (X.F.); a13876068647@163.com (J.A.); esther15208959100@163.com (P.Z.); 2 The University of Hong Kong Joint Laboratory of Tropical Infectious Diseases, Key Laboratory of Tropical Translational Medicine of Ministry of Education, School of Basic Medicine and Life Sciences, Hainan Medical University, Haikou 571199, China

**Keywords:** Human adenovirus, COVID-19 pandemic, hospitalized children, molecular typing, Hainan Island

## Abstract

Human adenoviruses (HAdVs) are significant viral pathogens associated with a wide spectrum of diseases, particularly acute respiratory tract infections (ARTIs) in children. This study aimed to characterize the epidemiological features of HAdV infections among hospitalized pediatric patients in Hainan Province, China, from January 2021 to December 2024, encompassing the COVID-19 pandemic and post-policy transition period. Among 31,843 hospitalized children with ARTIs, HAdVs were detected in 2464 cases (7.74%). Annual positivity rates were 4.87% in 2021, 7.21% in 2022, and 4.39% in 2023, and sharply increased to 11.58% in 2024. No significant sex-based difference in infection rates was observed. Distinctly seasonal and temporal patterns were noted, with a pronounced summer peak in 2024 and increased circulation throughout the year. A notable shift in dominant genotypes was observed, with HAdV-C initially prevailing, followed by a surge in HAdV-B between late 2023 and mid-2024. Children aged 1–7 years exhibited the highest positivity rates, indicating age-related susceptibility. Furthermore, co-infections were common and became increasingly predominant after the implementation of tNGS diagnostics, accounting for over 85% of HAdV-positive cases by 2023–2024. These findings suggest a post-pandemic resurgence of HAdV activity, shaped by altered population immunity, resumed social contact, and improved diagnostic sensitivity. Continuous molecular and epidemiological surveillance is essential to guide targeted interventions and inform pediatric respiratory infection management strategies.

## 1. Introduction

Human adenoviruses (HAdVs) are non-enveloped, double-stranded DNA viruses within the Adenoviridae family, which comprise more than 50 serotypes classified into seven species (A–G) [1,2,3]. These viruses are important etiological agents of a wide range of clinical syndromes, particularly acute respiratory tract infections (ARTIs) in children [4,5]. While most HAdV infections are self-limiting, certain serotypes—especially those within species B and C—are associated with severe lower respiratory tract disease, hospitalization, and even fatal outcomes, particularly in immunocompromised individuals [6,7,8]. In China, HAdV-B3 and HAdV-B7 have been frequently implicated in pediatric respiratory outbreaks requiring hospitalization [9,10].

During the coronavirus disease 2019 (COVID-19) pandemic, non-pharmaceutical interventions (NPIs)—including mask mandates, lockdowns, travel restrictions, and social distancing—significantly suppressed the circulation of many common respiratory viruses such as influenza virus, respiratory syncytial virus (RSV), and human metapneumovirus (hMPV) [11,12,13,14]. However, although HAdV circulation also declined during this period, the reduction was less pronounced than that observed for other respiratory viruses, likely reflecting their environmental stability and multiple transmission routes, including respiratory droplets, fecal-oral spread, and fomite contamination [15,16]. These public health interventions therefore substantially altered the epidemiological landscape of respiratory infections, including HAdVs, by reducing exposure opportunities and disrupting transmission chains, while not completely interrupting adenovirus circulation.

Following the large nationwide COVID-19 epidemic in early 2020, China entered a phase of normalized prevention and control in May 2020. In August 2021, a stringent Dynamic Zero-COVID strategy was adopted and maintained until 7 December 2022, when the zero-COVID policy was lifted in response to the emergence of Omicron subvariants with reduced pathogenicity and increasing vaccination coverage [17,18,19]. In Hainan Island, a tropical island located in southern China, experienced two major local SARS-CoV-2 waves in March–April 2022 and August–September 2022, during which stringent NPIs-including mass testing, mandatory case isolation, contact tracing, travel restrictions, and social distancing-were rigorously enforced. These public health interventions substantially altered the epidemiological landscape of other respiratory pathogens, including HAdVs, by reducing exposure opportunities, disrupting transmission chains, and potentially inducing “immunity debt” in young children [9,20,21].

Following the relaxation of COVID-19 control measures, the circulation of HAdVs demonstrated significant changes both in magnitude and pattern. In particular, emerging data from Hainan revealed a sharp resurgence of HAdV infections in 2024, surpassing pre-pandemic levels. Furthermore, a distinct genotype shift was observed, with HAdV-C predominating in mid-2022 to early 2023, followed by a rise in HAdV-B types as the dominant strains from late 2023 onward. These shifts in genotype dynamics may reflect changes in population-level immunity or viral fitness in the post-pandemic context.

Notably, the introduction of targeted next-generation sequencing (tNGS) in mid-2022 significantly improved the capacity to detect co-infections involving HAdV and other respiratory pathogens. As a result, co-infection rates rose markedly, reaching over 85% of HAdV-positive cases by 2023–2024. Meanwhile, seasonal patterns in Hainan—characterized by year-round transmission and summer peaks—differ markedly from those in temperate regions, likely influenced by the island’s tropical monsoon climate of high humidity and temperature.

Given these observations, we aimed to comprehensively investigate the temporal, demographic, molecular, and clinical characteristics of pediatric HAdV infections in Hainan Province across the COVID-19 pandemic and the subsequent post-pandemic period. This study aimed to analyze the epidemiological characteristics of HAdV among 31,843 hospitalized children with ARTIs in Hainan Province from 2021 to 2024, with a particular focus on shifts following the termination of the zero-COVID policy. The findings provide important insights into HAdV transmission dynamics in tropical regions and offer evidence to support more targeted prevention and control strategies for pediatric respiratory infections in the post-COVID era.

## 2. Methods

### 2.1. Sample Collection and HAdV Detection

Between January 2021 and December 2024, this study analyzed 31,843 samples from hospitalized children with ALRTIs at the Hainan Women and Children’s Medical Center. The research cohort comprised patients aged 18 years or younger, all presenting with symptoms indicative of ALRTIs upon hospital admission. Nasopharyngeal swabs were systematically collected from pediatric patients at admission to a standardized protocol and stored at −80 °C until testing for respiratory pathogens, including HAdVs. During this study period, the respiratory pathogen assay was progressively improved. From January 2021 to May 2022, respiratory pathogens were identified using a multiplex PCR assay combined with capillary electrophoresis analysis (KingMed Diagnostics, Guangzhou, China) targeting 18 common respiratory pathogens. This panel included major respiratory viruses and atypical/typical bacteria, such as respiratory syncytial virus, rhinovirus, adenovirus, human parainfluenza viruses, human coronaviruses, influenza A (including A/H3N2 and A/pdmH1N1(2009)) and influenza B viruses, bocavirus, *Mycoplasma pneumoniae*, *Chlamydia* spp., *Haemophilus influenzae*, *Streptococcus pneumoniae*, *Legionella pneumophila*, *Mycobacterium tuberculosis*, and *Bordetella pertussis* [22]. From June 2022 to December 2024, the assay was converted to a tNGS panel (KingMed Diagnostics, Guangzhou, China) as previously described [23,24,25]. This tNGS panel was designed to target conserved genomic regions of 153 respiratory pathogens, covering more than 95% of known respiratory infections, and was capable of detecting 65 bacterial species, 68 viruses (25 DNA viruses and 43 RNA viruses), 14 fungi, and 6 *mycoplasmas*/*chlamydiae*. In the present study, an HAdV infection was defined as detection of HAdV nucleic acid by either platform, and HAdV co-infection was defined as detection of HAdV together with at least one additional pathogen on the same multiplex PCR or tNGS panel.

### 2.2. Statistical Analysis

Data were analyzed using the χ^2^ test with SPSS software (version 25.0). Statistical significance was set at *p* < 0.05. Figures were created using Origin 2021.

## 3. Results

### 3.1. Sample Information and Demographics

From January 2021 through December 2024, a total of 31,843 pediatric inpatients diagnosed with ARTIs were admitted to Hainan Women and Children’s Medical Center. The demographic characteristics are summarized in Table 1. Among these patients, 19,335 were male (60.72%) and 12,508 were female (39.28%), with a male-to-female ratio of 1.55:1. Annual distributions were as follows: 4419 cases (13.88%) in 2021, 5188 (16.29%) in 2022, 9732 (30.56%) in 2023, and 12,504 (39.27%) in 2024. The age range of the children was 0–18 years, with a median age (interquartile range) of 2.38 (0.82–4.92) years.

The overall HAdV-positive rate was 7.74% (2464/31,843), with annual positivity rates of 4.87% (215/4419) in 2021, 7.21% (374/5188) in 2022, 4.39% (427/9732) in 2023, and 11.58% (1448/12,504) in 2024. Among HAdV-positive cases, 61.61% (1518/2464) were male and 38.39% (946/2464) were female. Although males outnumbered females across all four years, the difference was not statistically significant (χ^2^ = 0.882, *p* = 0.348), suggesting no gender-specific susceptibility.

### 3.2. Monthly Incidence Trends Before and After the Termination of Dynamic Zero-COVID Policy

**Figure 1:**
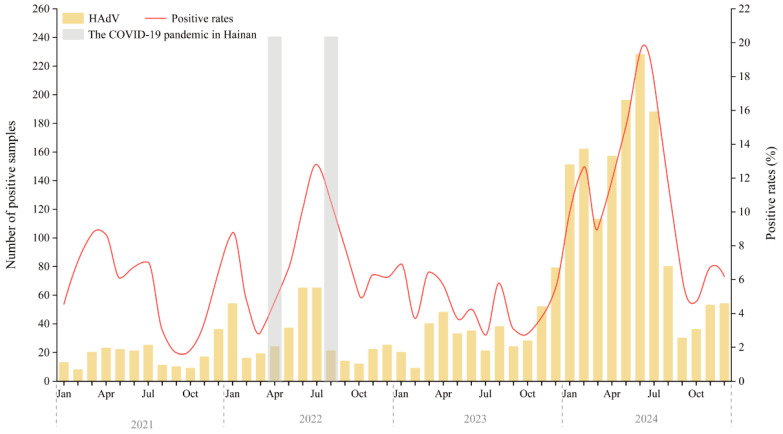
Annual Trends and Monthly Distribution of HAdV Infections Among Hospitalized Children in Hainan (2021–2024). The gray-shaded areas indicate the two major local SARS-CoV-2 epidemic waves on Hainan Island (March–April 2022 and August–September 2022), which occurred under the national Dynamic Zero-COVID policy that remained in effect until 7 December 2022. Abbreviations: Jan, January; Apr, April; Jul, July; Oct, October.

Figure 1 displays the monthly distribution of HAdV-positive cases and positivity rates from January 2021 to December 2024. Compared to 2021 and 2022, both the number of tests performed and the number of HAdV-positive cases increased in 2023. However, the positivity rate in 2023 (4.39%) was lower than in 2021 (4.87%) and 2022 (7.21%).

Marked peaks in HAdV activity occurred in February–July and December of 2021, and January, May–September, and November–December of 2022. In 2023, activity was more evenly distributed, with smaller peaks in January, March-April, August, and December. A sharp resurgence was observed in 2024, with cases rising steadily from January and peaking in June at over 220 cases and a positivity rate of 19.42%—the highest in the surveillance period.

### 3.3. Seasonal Distribution of HAdV-Positive Cases

**Figure 2:**
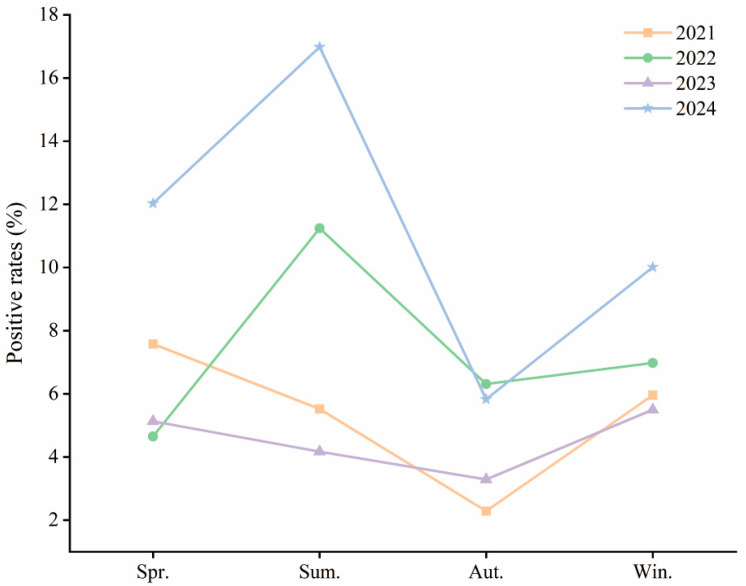
Annual HAdV Infection Rates by Season Among Hospitalized Children in Hainan (2021–2024). Abbreviations: Spr., Spring; Sum., Summer; Aut., Autumn; Win., Winter. Spring: March–May; Summer: June–August; Autumn: September–November; Winter: December–February.

Seasonal patterns varied significantly over the years. For the purposes of this study, spring was defined as March–May, summer as June–August, autumn as September–November, and winter as December–February. In 2021, positivity rates were higher in spring (7.58%), summer (5.52%), and winter (5.96%), with autumn being the lowest (2.29%) (χ^2^ = 39.512, *p* < 0.001). In 2022, summer showed the highest positivity (11.24%), with significant seasonal differences (χ^2^ = 50.513, *p* < 0.001). In 2023, spring and winter had higher rates than autumn (χ^2^ = 18.190, *p* < 0.001). In 2024, positivity peaked in summer (16.98%), followed by spring (12.03%) and winter (10.01%) (χ^2^ = 158.75, *p* < 0.001) (Table 1, Figure 2).

The seasonal shift suggests that HAdV has transitioned to a year-round circulation pattern with summer predominance in recent years, likely influenced by Hainan’s tropical climate, increased post-pandemic social interaction, and waning immunity in children.

### 3.4. Molecular Typing and Seasonal Distribution of HAdV Species

**Figure 3:**
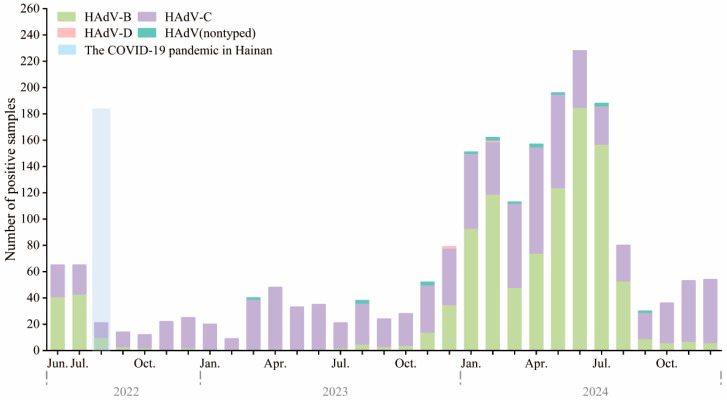
Monthly distribution of HAdV types among hospitalized children in Hainan, China, from June 2022 to December 2024. Stacked bar chart showing the monthly number of HAdV-positive samples, stratified by type, including HAdV-B (green), HAdV-C (purple), HAdV-D (pink), and untyped HAdV (teal). The light blue shaded area indicates the second major local SARS-CoV-2 epidemic wave on Hainan Island (August–September 2022), which occurred under the Dynamic Zero-COVID policy that ended on 7 December 2022. In total, 224, 427, and 1448 HAdV-positive samples were genotyped in 2022 (June–December), 2023, and 2024, respectively.

Between June 2022 and December 2024, HAdV-positive samples were analyzed using a tNGS respiratory pathogen panel. In mid-2022 to mid-2023, HAdV-C (purple) was the predominant type, with fewer than 40 monthly cases. HAdV-B (light green) and HAdV-D (pink) were rarely detected (Figure 3).

A surge began in late 2023, peaking between January and July 2024, with monthly counts exceeding 240. This was driven by a dominance shift to HAdV-B, which replaced HAdV-C as the main circulating type. HAdV-B peaked in spring and early summer (March–June), then sharply declined. HAdV-C persisted year-round at lower levels. HAdV-D and untyped cases were rare. This apparent change in genotype predominance, especially the emergence of HAdV-B during post-COVID policy relaxation, may be related to changes in population immunity or viral fitness.

### 3.5. Co-Infection Patterns

**Figure 4:**
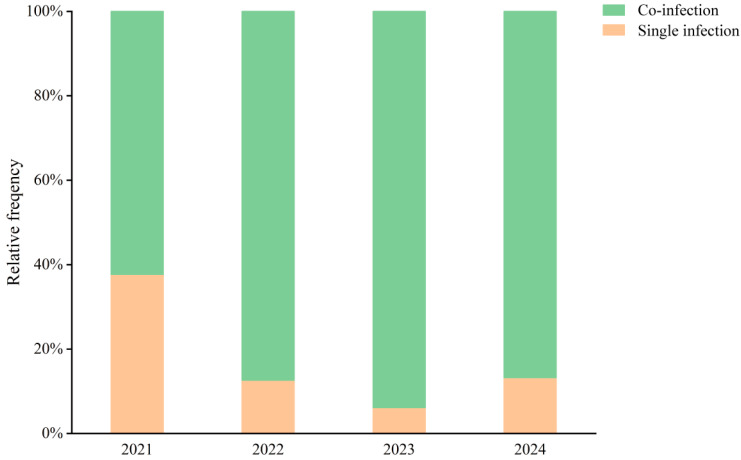
Co-infection Rates of HAdV and Other Respiratory Pathogens Among Hospitalized Children in Hainan (2021–2024). Stacked bar chart illustrating the relative frequency (%) of single infections (orange) and co-infections (green) with adenovirus detected in pediatric patients during the years 2021–2024.

Figure 4 illustrates that co-infections consistently accounted for a higher proportion of cases than single infections throughout the study period. In 2021, 62% of HAdV-positive cases involved co-infections. Following the introduction of tNGS in June 2022, the proportion of co-infections rose markedly—reaching 88% in 2022 and 94% in 2023. In 2024, co-infections remained dominant, accounting for 87% of all HAdV-positive cases. These findings likely reflect both a broader resurgence of respiratory viruses and improved detection capacity through comprehensive multiplex testing.

Although not shown in the figure, the annual proportion of severe HAdV-associated cases also increased, from 0.18% (8/4419) in 2021 to 0.50% (62/12,504) in 2024 (χ^2^ test, *p* = 0.008; 95% CI for the difference: 0.06–0.39 percentage points). This numerical increase parallels rising co-infection rates; however, it should be interpreted cautiously, as it may partly reflect methodological differences between the multiplex PCR and tNGS phases and should be regarded as a descriptive association rather than evidence of a direct causal effect of co-infections on disease severity. These results highlight the importance of multiplex diagnostic platforms in early pathogen identification and clinical risk stratification for pediatric patients with respiratory infections.

### 3.6. Age-Based Analysis

Children were stratified into four age groups: infants (0–1 year), toddlers (1–3 years), preschoolers (3–7 years), and school-age (7–18 years) (Table 1). The highest positivity rates occurred in children aged 3–7 years, peaking at 15.39% in 2024, followed by toddlers, with rates of 9.44% in 2022 and 14.2% in 2024. Infants and school-age children consistently showed lower rates.

In 2024, all age groups experienced a notable rise in infection rates, particularly the 1–7-year cohort, which may indicate increased vulnerability or exposure in the post-pandemic period. Notably, the positivity rate in the 7–18-year group rose from <4% to over 11% in 2024, implying a shift in age-related susceptibility.

These findings underscore the need for targeted surveillance and prevention strategies in kindergartens and primary schools, where transmission risk is highest.

## 4. Discussion

This large-scale surveillance study provides a comprehensive overview of HAdV infections among hospitalized children in Hainan Province from 2021 to 2024, encompassing the transition from the COVID-19 pandemic to the post-policy era. The findings reveal marked changes in the epidemiological landscape of HAdV infections following the termination of China’s Dynamic Zero-COVID strategy, characterized by a sharp resurgence in 2024, significant age-related differences, an apparent change in the predominant circulating genotypes during the study period, and a marked rise in co-infections. Because our hospital serves as the main tertiary referral center for children in Hainan Province, this cohort provides a comprehensive picture of the burden and clinical spectrum of HAdV-associated ARTIs requiring hospitalization in this tropical island setting.

In particular, the sharp surge in HAdV cases observed in 2024 likely reflects a post-pandemic rebound in respiratory viral transmission following the relaxation of strict NPIs, restoration of population mobility, and the presence of immunity gaps in children. Similar delayed epidemics and intensified circulation have been documented for other pediatric respiratory viruses after the lifting of COVID-19 control measures, supporting the concept of ‘immunity debt’ and the role of renewed social mixing in driving viral resurgence in this age group [21,26,27].

Over the entire four-year period, the overall HAdV positivity rate among children hospitalized with ARTIs was 7.74%, with marked inter-annual variation. During 2021–2023, positivity ranged from 4.39% to 7.21% despite substantial increases in testing volume, indicating that HAdV maintained endemic circulation even under stringent Dynamic Zero-COVID measures. In 2024, the positivity rate rose to 11.58%, representing an almost two-fold increase compared with the preceding three years. Notably, both the absolute number of tests and the proportion of positives increased, suggesting a genuine intensification of community transmission rather than a testing artifact. These findings are consistent with a post-policy rebound of respiratory virus activity in a pediatric population with accumulated susceptibility, and they complement our age-stratified and genotype-specific analyses of the 2024 surge [28,29].

Our analysis demonstrated that children aged 1–7 years exhibited the highest HAdV positivity rates, consistent with prior studies suggesting that immature immune systems increase vulnerability to adenoviral infections [4,26]. A notable feature in 2024 was the sharp increase in positivity among school-age children (7–18 years), which may reflect an “immunity debt”—a reduction in population-level immunity due to limited viral exposure during prolonged NPIs. Similar patterns have been reported in other respiratory viruses, supporting the hypothesis that relaxed restrictions and renewed social interactions contributed to widespread viral resurgence in pediatric populations [21,26]. These findings emphasize the need for enhanced surveillance and preventive measures in kindergartens and schools, where close contact facilitates viral transmission.

Distinct seasonal dynamics were observed, with consistently elevated activity in spring, summer, and winter, and lower detection in autumn. This year-round circulation contrasts with reports from temperate regions such as northern China and Europe, where HAdV infections peak mainly during winter and early spring [30,31]. Hainan’s tropical monsoon climate, characterized by high temperature and humidity, likely sustains viral transmission throughout the year, producing a unique epidemiological pattern dominated by summer peaks. Additionally, we observed an apparent transition in the predominant detected genotypes, from HAdV-C (more frequently identified in 2022 to early 2023) to HAdV-B (more frequently identified in late 2023 to mid-2024), which may reflect transient changes in viral adaptation or host immunity; however, longer-term surveillance is needed to determine whether this represents a sustained shift in genotype dominance [26,29]. These findings underscore the importance of region-specific public health strategies tailored to local climatic and social conditions.

Our findings are also consistent with emerging national data indicating a broader resurgence of HAdV activity in China after the lifting of Dynamic Zero-COVID policies [27]. Recent reports from pediatric and sentinel surveillance cohorts in other provinces have documented increased HAdV detection and outbreaks since 2023–2024, frequently involving shifts in predominant genotypes and atypical seasonal patterns in children [28,30]. These studies suggest that HAdV circulation, which was substantially suppressed during periods of stringent COVID-19 control, has rebounded across multiple regions in the post-policy era, with a growing contribution from species B viruses [31]. Taken together, these observations indicate that the resurgence and genotype shift observed in Hainan form part of a wider, country-level pattern of intensified HAdV circulation in pediatric populations, while also highlighting the distinctive influence of the tropical island climate on local transmission dynamics.

Co-infections were highly prevalent, rising from 62% in 2021 to over 85% after the introduction of tNGS in mid-2022, reaching 94% in 2023. The substantial increase may be attributed to both improved diagnostic resolution and ecological shifts in respiratory pathogen circulation following the end of pandemic restrictions. In particular, the transition from multiplex PCR to a more sensitive tNGS platform likely enhanced the detection of low-abundance and multi-pathogen infections, and thus may have contributed to higher apparent co-infection rates in the later years. Similarly, the parallel increase in severe HAdV-associated cases should be interpreted cautiously, as it may partly reflect methodological and case-mix differences between study phases rather than a direct causal effect of co-infections on disease severity. Co-infections involving HAdV are clinically significant, as viral–bacterial or viral–viral interactions can exacerbate inflammation, prolong illness, and increase disease severity [32,33]. The parallel increase in severe HAdV-associated cases from 0.18% in 2021 to 0.50% in 2024 is consistent with this descriptive association but should be interpreted cautiously and does not establish a causal relationship between co-infections and disease severity. These results highlight the crucial role of multiplex diagnostic platforms in early identification, risk stratification, and clinical management of pediatric ARTIs.

The observed post-pandemic resurgence of HAdV infections illustrates how abrupt policy transitions can reshape viral ecology. The relaxation of strict NPIs, increased population mobility, and waning immunity collectively created a permissive environment for viral transmission [27]. Continuous molecular surveillance is essential to detect emerging HAdV genotypes, monitor co-infection dynamics, and assess the clinical burden of severe disease [34,35]. Furthermore, understanding genotype-phenotype associations may guide the development of effective adenovirus vaccines, which remain an unmet need for pediatric populations worldwide [36].

This study has several limitations. It was conducted at a single tertiary pediatric center, which may limit generalizability to broader regions. As a provincial referral women and children’s medical center, our hospital predominantly manages hospitalized children with clinically significant ARTIs, including many moderate-to-severe or complicated cases. Consequently, the HAdV positivity rates, co-infection patterns, and severe case burden described here are more reflective of the epidemiology of HAdV infections among hospitalized patients than of community-based or primary-care populations, and may overestimate the severity profile compared with milder outpatient infections. Second, the transition from multiplex PCR to tNGS during the study period may have introduced temporal variation in diagnostic sensitivity. This change could inflate the observed co-infection rates and complicate direct comparisons of pathogen spectra and severity between the pre- and post-tNGS phases, potentially leading to overestimation of co-infection burden in the later years. In addition, although tNGS provides high sensitivity and broad coverage, it may still fail to capture untyped or recombinant strains. Future research should involve multicenter longitudinal studies, whole-genome sequencing, and host immune profiling to elucidate viral evolution, host–pathogen interactions, and the mechanisms underlying disease severity. Integrating environmental data such as temperature, humidity, and air quality could further clarify how climate modulates HAdV transmission in tropical regions.

## 5. Conclusions

In summary, this study elucidates the evolving epidemiology of HAdV infections among children in Hainan Province during and after the COVID-19 pandemic. The findings highlight the synergistic influence of climate, host age, viral genotype, and public health policies on infection dynamics. Sustained surveillance, combined with adaptive prevention and control strategies, is crucial to mitigating the burden of adenoviral and other respiratory pathogens in pediatric populations in the post-pandemic era.

## Figures and Tables

**Table 1:** Demographic Characteristics of HAdV-Positive Cases from 2021 to 2024.

Characteristic		Total (*n* = 31,843)	2021 (*n* = 4419)	2022 (*n* = 5188)	2023 (*n* = 9732)	2024 (*n* = 12,504)
Gender	Male	1518 (7.85%)	135 (4.81%)	242 (7.68%)	266 (4.47%)	875 (11.78%)
	Female	946 (7.56%)	80 (4.97%)	132 (6.47%)	161 (4.26%)	573 (11.28%)
χ^2^		0.882	0.059	2.714	0.247	0.733
P		0.348	0.809	0.099	0.619	0.392
Age	0–1	341 (3.55%)	39 (2.15%)	43 (3.05%)	61 (2.27%)	198 (5.35%)
	1–3	773 (9.79%)	85 (6.51%)	122 (9.44%)	154 (6.44%)	412 (14.18%)
	3–7	1082 (10.61%)	87 (8.24%)	182 (10.14%)	166 (5.29%)	647 (15.39%)
	7–18	268 (6.47%)	4 (1.67%)	27 (3.89%)	46 (3.03%)	191 (11.27%)
χ^2^		410.194	67.900	80.518	65.336	218.798
P		0.000	0.001	0.000	0.000	0.000
Season	Spring	732 (8.31%)	65 (7.58%)	80 (4.65%)	121 (5.13%)	466 (12.03%)
	Summer	798 (10.57%)	57 (5.52%)	151 (11.24%)	94 (4.17%)	496 (16.98%)
	Autumn	307 (4.08%)	36 (2.29%)	48 (6.31%)	104 (3.29%)	119 (5.83%)
	Winter	627 (7.89%)	57 (5.96%)	95 (6.98%)	108 (5.50%)	367 (10.01%)
χ^2^		230.582	39.512	50.513	18.190	158.759
P		0.000	0.000	0.000	0.000	0.000

## Data Availability

The original contributions presented in the study are included in the article, further inquiries can be directed to the corresponding authors.
